# Platelet-rich fibrin promotes mesothelial cell proliferation and peritoneal repair by up-regulating calretinin to prevent postoperative intestinal adhesion

**DOI:** 10.7150/ijms.105523

**Published:** 2025-02-18

**Authors:** Xinming Li, Yifan Guo, Zhuoyin Wang, Xu Guo, Jia Wang, Jianlu Zhang, Tao Zhang, Jing Wang, Tianxiong Li, Jian Zhou, Nengwei Zhang, Buhe Amin, Bin Zhu

**Affiliations:** 1 Department of General Surgery, Beijing Shijitan Hospital, Capital Medical University, Beijing, 100038, China.; 2 Department of Thoracic Surgery, Beijing Haidian Hospital, Haidian Section of Peking University Third Hospital, Beijing, 100080, China.; 3 Department of Thoracic Surgery, Beijing Shijitan Hospital, Capital Medical University, Beijing, 100038, China.; 4 Department of General Surgery, Peking University Third Hospital, Beijing, 100191, China.; 5 Department of General Surgery, Peking Union Medical College Hospital, Chinese Academy of Medical Sciences and Peking Union Medical College, Beijing, 100730, China.; 6 Department of Endocrinology, Beijing Liangxiang Hospital, Capital Medical University, Beijing, 102401, China.; 7 Department of Science and Technology, Beijing Shijitan Hospital, Capital Medical University, Beijing, 100038, China.

**Keywords:** PRF, intestinal adhesion, mesothelial cell, fibroblast, CR

## Abstract

**Introduction:** As the most common postoperative complication, intestinal adhesions can cause intestinal obstruction, female infertility, and even endanger life. The currently developed materials for preventing intestinal adhesions mainly focus on physical barriers and reducing inflammatory reactions, while neglecting the importance of effectively promoting rapid repair of the peritoneum. We previously found that platelet-rich fibrin (PRF) can prevent postoperative intestinal adhesions. The proliferation of mesothelial cells may play a significant role in reducing intestinal adhesions, but the mechanism remains unclear. A study found a positive correlation between calretinin (CR) and mesothelial cell proliferation. Does CR play an important role in PRF promoting mesothelial cell proliferation? This study aims to further explore the mechanism of PRF in preventing intestinal adhesions.

**Methods:** Primary mouse peritoneal mesothelial cells and mouse peritoneal fibroblasts were used in this study. The effects of PRF on the proliferation and attachment of mesothelial cells and fibroblasts were observed and compared using the CCK-8 assay, Edu assay, and laser scanning confocal microscope. The effects of PRF on the migration of mesothelial cells were examined using scratch and transwell migration assays. The effects of PRF on the mesothelial-mesenchymal transition (MMT) of mesothelial cells were examined using western blot. The expression level of CR in mesothelial cells was detected through immunofluorescence, quantitative real-time polymerase chain reaction (qRT-PCR), and western blot.

**Results:** PRF promotes mesothelial cell proliferation from 1st day and significantly promotes fibroblast proliferation from 7th day. Meanwhile, PRF tends to promote the proliferation and attachment of mesothelial cells rather than fibroblasts. However, PRF had a limited regulatory effect on the MMT of mesothelial cells. In addition, PRF can promote mesothelial cell migration and upregulate the expression level of CR.

**Conclusion:** PRF promotes mesothelial cell proliferation and migration, as well as peritoneal repair, by up-regulating CR in the early stages of peritoneal injury to prevent postoperative intestinal adhesion. Its mechanism is obviously different from that of traditional anti-adhesion materials, which will provide new strategies for the prevention of intestinal adhesions.

## 1. Introduction

Abdominal adhesions (including intestinal adhesions) are usually caused by multiple reasons such as abdominal surgery, trauma, and peritonitis 1-3. The incidence of postoperative abdominal adhesions is as high as 79% -90% 4,5. In addition to causing intestinal obstruction, female infertility, and chronic pelvic and abdominal pain, abdominal adhesions can also increase the difficulty of abdominal reoperation and can be life-threatening in severe cases 6-8.

The mechanism of abdominal adhesion formation is complex and not yet fully understood. The formation of abdominal adhesions may be the result of a combination of inflammatory reactions, coagulation, fibrinogen production, and extracellular matrix (ECM) deposition 9. Neutrophil extracellular traps (NETs), macrophage superaggregates, mast cells, T lymphocytes, fibroblasts, and mesothelial cells are involved in this process and play important roles in adhesion formation 10. At present, the materials, drugs, and other methods developed to prevent abdominal adhesions are far from meeting clinical needs in terms of effectiveness and safety 11,12. Finding efficient and low-side-effect materials for preventing intestinal adhesions has always been an urgent problem in clinical practice.

Research has found that the occurrence and degree of abdominal adhesions are not only related to peritoneal injury but also closely related to the repair speed of the peritoneum after peritoneal injury. When a continuous mesothelial cell layer is formed in the early stage of peritoneal injury, the degree of adhesions is significantly reduced even not adhesion 13,14. As the main barrier of the abdominal cavity, Peritoneal mesothelial cells may play an important role in the occurrence and development of adhesion 15-17. Research has found that after peritoneal injury, mesothelial cells undergo mesothelial-mesenchymal transition (MMT) and participate in the formation of pathological abdominal adhesions 18, 19. However, the materials currently used in clinical practice for preventing adhesions mainly focus on physical barriers and reducing inflammatory reactions to prevent intestinal adhesions, neglecting the importance of effectively promoting rapid repair of the peritoneum. Therefore, we hypothesize whether a certain material can achieve the effect of preventing and reducing intestinal adhesions by promoting the proliferation of mesothelial cells and rapid repair of the peritoneum?

Platelet-rich fibrin (PRF) belongs to the second generation of platelet concentrate products, which are rich in various growth factors and can promote cell proliferation and accelerate wound healing 20. Our previous studies have shown that PRF is rich in transforming growth factor-β (TGF-β), vascular endothelial growth factor (VEGF), platelet-derived growth factor (PDGF), epidermal growth factor (EGF), and other growth factors, which can accelerate the healing of pressure sore wounds in rats and skin wounds in diabetes mice by promoting cell proliferation 21, 22. In recent years, PRF has been used for the repair of bone and oral wounds and has achieved a satisfactory therapeutic effect 23-25.

More importantly, we first discovered that PRF can prevent postoperative intestinal adhesions in previous animal experiments 26. The proliferation of mesothelial cells may play an important role in reducing intestinal adhesions, but its mechanism is still unclear. A study has found a positive correlation between calretinin (CR) and mesothelial cell proliferation 27. Does CR play an important role in PRF promoting mesothelial cell proliferation? Meanwhile, does PRF have an impact on MMT?

Primary mouse peritoneal mesothelial cells and mouse peritoneal fibroblasts were used in this study to investigate the effects of PRF on mesothelial cell proliferation, attachment, migration ability, and MMT. Meanwhile, we preliminarily explored the possible molecular mechanism of PRF promoting mesothelial cell proliferation through changes in CR expression level. To the best of our knowledge, this is the first study investigating the effects of PRF on mouse peritoneal mesothelial cells.

## 2. Materials and Methods

### 2.1 Experimental animal

60 male inbred strain BALB/C mice (4-6 weeks old, 18-20 g of body weight) were obtained from Beijing Vital River Laboratory Animal Technology Co., Ltd. Among them, 40 mice were used for the preparation of PRF, and 20 mice were used for the isolation of peritoneal mesothelial cells and fibroblasts. The project was approved by the Institutional Animal Care and Use Committee at Beijing Shijitan Hospital, Capital Medical University, China (2018 No. 69 Research Ethics).

### 2.2 Isolation and identification of primary mouse peritoneal mesothelial cells

Primary mouse peritoneal mesothelial cells were isolated by intraperitoneal injection as previously described 28. Briefly, after euthanizing the mice, 5mL of 0.25% trypsin/ EDTA solution (Gibco, USA) was rapidly injected into the abdominal cavity. The mice were placed in a supine position and a prone position respectively for 5 minutes. The abdominal digestion solution containing isolated cells was collected and centrifuged at 1000rpm for 10min in a low-speed centrifuge (Thermo Fisher Scientific, USA). Use PBS (Gibco, USA) containing 4%penicillin-streptomycin (Gibco, USA) to wash the cell precipitation and add it to mesothelial cell-specific complete culture medium composed of DMEM/F12 (Solarbio, China), 10% FBS (Solarbio, China), 10ng/ml epidermal growth factor (EGF, Procell, China),0.4μg/ml hydrocortisone (Procell, China), 10ng/ml epidermal growth factor, 1% insulin, transferrin, selenium (ITS, Procell, China) and 1% Penicil-lin-Streptomycin as previously described 29. Culture is in a cell incubator (37 °C, 5% CO2, 95% air) and the medium was replaced 24h later at first and then replaced every 3 days. Cells were cultured for about 5-7 days until they were at 80%-90% confluency. A previous method based on different adhesion rates of cell types was applied to purify mesothelial cells, specifically the principle that mesothelial cells adhere to surfaces at a slower rate than fibroblasts as previously described 30. Briefly, the isolated cells (predominantly consisting of mesothelial cells and fibroblasts) are inoculated in a cell culture dish and incubated for a suitable duration (20 minutes). Fibroblasts gather on the bottom wall of the dish due to their faster adhesion time, while mesothelial cells with slower adhesion time are predominantly found in the culture medium. The medium containing the mesothelial cells is then transferred to a new cell culture dish for continued cultivation. This process is repeated to achieve effective separation of mesothelial cells from fibroblasts, thereby purifying the mesothelial cell population. When the growth and fusion of the purified mesothelial cells (2nd generation) reached 90%, the morphology of the mesothelial cells was observed under an inverted microscope (Shanghai, Cai Kang). Cytokeratin antibody (Bioss, bsm-34137M, 1:100 diluted, China), vimentin antibody (Bioss, bs-0756R, 1:100 diluted, China), and Alpha smooth muscle actin(α-SAM) antibody (Bioss, bsm-33188M, 1:100 diluted, China)were selected to identify the mesothelial cells.

### 2.3 Isolation and identification of primary mouse peritoneal fibroblasts

Primary mouse peritoneal fibroblast cells were isolated from the peritoneum as previously described 31. Briefly, after euthanizing the mice, soak them in 75% alcohol for 10min. The peritoneal cavities were then opened and the entire peritoneum was isolated under sterile conditions. The peritoneum was minced using a sterile scalpel into 1 mm^3^ pieces. Use PBS containing 4% penicillin-streptomycin to wash the peritoneum tissue block and add it to an appropriate amount of DMEM complete culture medium (Solarbio, China) containing 10% FBS and 1% penicillin-streptomycin to resuspend the wall peritoneum tissue block in the EP tube. Evenly inoculate it into a cell culture dish and invert it in the cell incubator for 3h to ensure complete attachment of the tissue block to the wall. Then cells were cultured with DMEM complete culture medium and the medium was replaced every 3 days. When the fibroblasts grew from the tissue block and fused into a dense monolayer (usually 7-10 days), they were digested with 0.25% trypsin and filtered through a 70μm cell sieve (Corning, USA). Then they were cultured after centrifuging at 1000rpm for 5min. When the growth and fusion of fibroblasts reached 90%, the morphology of fibroblasts was observed under an inverted microscope. Alpha smooth muscle Actin(α-SAM) antibody (Bioss, bsm-33188M, 1:100 diluted, China), vimentin antibody (Bioss, bs-0756R, 1:100 diluted, China), and cytokeratin antibody (Bioss, bsm-34137M, 1:100 diluted, China) were selected to identify the fibroblasts.

### 2.4 Preparation of PRF gel, film and leachate

**Figure 1:**
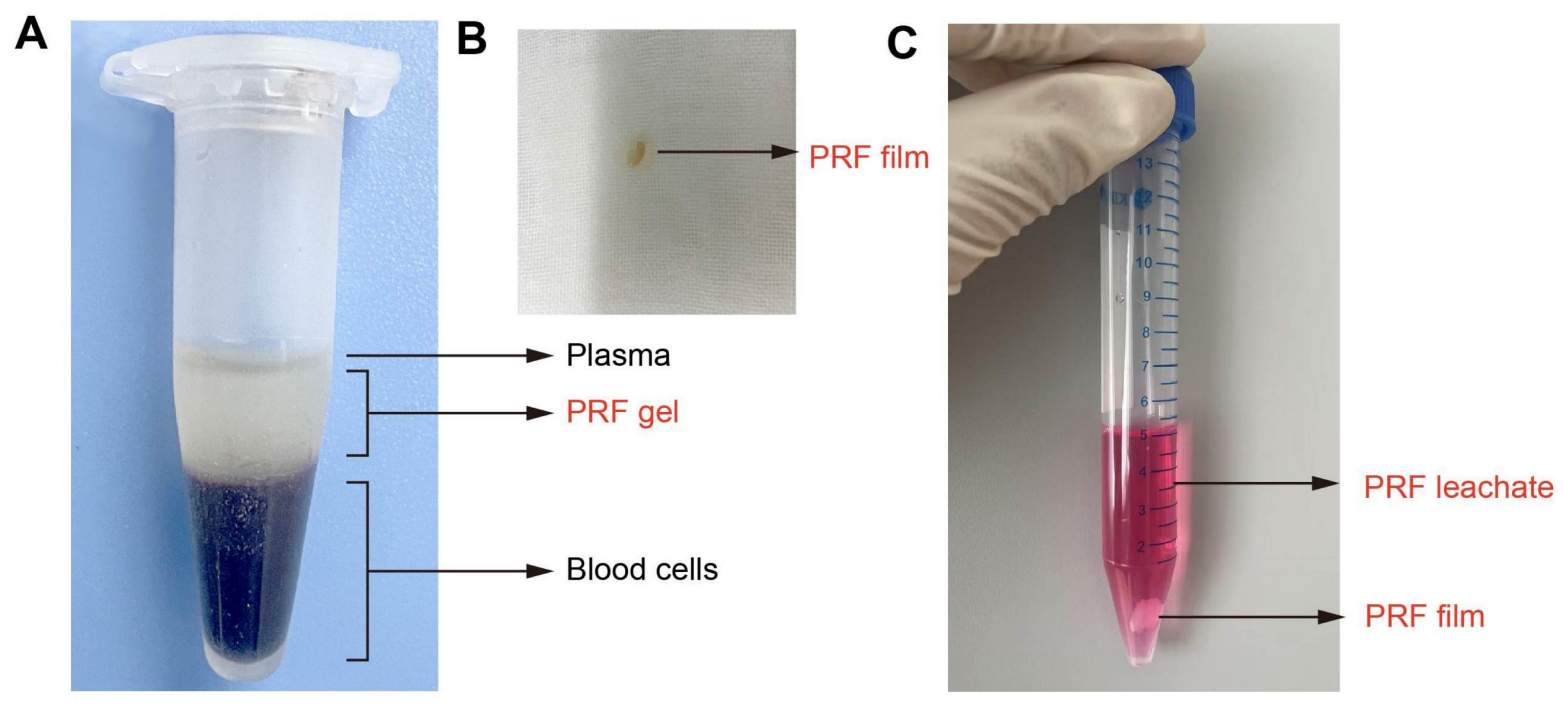
** Preparation of PRF gel, film, and leachate.** (A) The intermediate layer after centrifugation is PRF gel. (B) PRF film. (C) PRF leachate.

The PRF film is made by extruding PRF gel with sterile gauze as described in our previous animal experiments 26. Choukroun's method 20 was used to prepare PRF gel, and the entire operation strictly followed the principle of sterility. Briefly, use sterile instruments to remove one side of the mouse eyeball and take 0.5-1.0mL of blood in a 1.5mL EP tube. Centrifuge the EP tube at 3000rpm for 10min. After standing for 5min, the blood in the EP tube was divided into three layers, which are plasma, PRF gel, and red blood cells from top to bottom, and the PRF gel in the middle layer is gelatinous (Figure 1A). Take out the gelatinous substance when cutting off the bottom layer containing red blood cells with Schwann's scissors and suck out the excess serum. The gelatinous substance is the PRF gel. Use sterile gauze to squeeze the PRF gel into a PRF film with a thickness of 1mm (Figure 1B). PRF leachate is made by immersing PRF film in a complete culture medium as previously described 24. In summary, a PRF film was molded to dimensions of 1×1×1 mm³ and then incubated in 5 mL of either mesothelial cell-specific medium or DMEM complete medium. This mixture was maintained in a cell incubator for 7 days, after which the resulting supernatant was collected as the PRF leachate (Figure 1C). Define the concentration of PRF leachate taken out for the first time as 100% and dilute it with the corresponding ingredients of the culture medium into PRF leachate of different concentrations (volume fractions) of 20%, 40%, 60%, and 80%. Finally, store them in a refrigerator at -80 °C for standby.

### 2.5 Detection of cell proliferation ability

The following three experimental methods were applied to detect and compare the effects of PRF on the proliferation ability of cells.

#### 2.5.1 The CCK-8 assay was used to detect the effect of PRF on the proliferation ability of mesothelial cells and fibroblasts

According to whether mesothelial cells or fibroblasts and PRF leachate were added to the culture medium, they were divided into a control group (culture medium+cells), a PRF group (culture medium+cells+PRF), and a blank control (only culture medium) with three independent biological replicates in each group. To screen for the optimal concentration of PRF leachate, five concentrations of PRF leachate (20%, 40%, 60%, 80%, and 100%) were designated as PRF1, PRF2, PRF3, PRF4, and PRF5 respectively. Select the third-generation mesothelial cells or fibroblasts, adjust the cell concentration to 5×10^4^ cells/mL, and inoculate the cells into the 96-well plates (Corning, USA) according to grouping. The cell counting kit-8 (CCK-8, Beyotime, China) was used following the manufacturer's instructions. Briefly, prepare a complete culture medium containing 10% CCK-8 and aspirate the culture medium from the well plate. Add 1 ml of complete culture medium containing 10% CCK-8 to each well and continue incubating the culture plate in the incubator for 2h. The optical density (OD) at 450 nm was measured and recorded using a microplate reader (3001, Thermo Fisher Scientific, USA) at different time points (1d, 3d, 5d, and 7d of culture).

#### 2.5.2 Edu assay detects the effect of PRF on the proliferation ability of mesothelial cells

The appropriate concentration of PRF leachate was selected according to the CCK-8 assay. Mesothelial cells were digested and the cell concentration was adjusted to 5×10^4^ cells/mL so that cells were inoculated into the 24-well plates (Corning, USA) according to grouping. Divide mesothelial cells into a control group and a PRF group based on whether PRF was added or not. Each group was divided into three subgroups according to different culture times, with three independent biological replicates in each subgroup. After 24h, 48h, and 72h of culture, the percentage of positive cells (green) in each group was detected using the BeyoClick EdU Cell Proliferation Kit with Alexa Flouor 488 (Beyotime, China) and Hoechst33342 was used for nuclear staining according to the manufacturer's instructions. Cells stained with both green and blue were considered EdU-positive cells.

#### 2.5.3 Comparison of the effects of PRF on the proliferation ability of mesothelial cells and fibroblasts using laser scanning confocal microscopy

Mouse peritoneal mesothelial cells and fibroblasts were stained with cell membrane fluorescent dyes Dil (red, Beyotime, China) and Dio (green, Beyotime, China) respectively as previously described 32. Briefly, take 1μL of cell membrane dye Dil storage solution and Dio storage solution with a concentration of 5mM respectively, and add 1mL PBS to prepare a working solution with a concentration of 5μM. Mesothelial cells and fibroblasts were digested and the cell concentration was adjusted to 5×10^4^ cells/mL. Add Dil working solution and Dio working solution to the precipitation of mesothelial cells and fibroblasts respectively. Resuspend the cells and incubate them in a cell culture incubator for 20 min and then centrifuge them at 1000rpm for 5 min to obtain cell precipitation. After resuspending two types of cells in a complete culture medium, the cells were divided into three groups: mesothelial cell group, fibroblast cell group, and mesothelial cell fibroblast co-culture group in a 1:1 ratio. Inoculate cells into the 24-well plates according to grouping. Select the appropriate concentration of PRF leachate based on the CCK-8 assay, and divide it into the control group and PRF group according to whether PRF is added or not. Three independent biological replicates were assessed in each group. After incubating in the incubator for 1d, 3d, 5d, and 7d, the number and proportion of cells in each group were observed using a laser scanning confocal microscope (Nikon, Japan).

### 2.6 Comparison of the effects of PRF on the adhesion ability of mesothelial cells and fibroblasts using laser scanning confocal microscopy

The mesothelial cells and fibroblasts were digested and the cell concentration was adjusted to 1×10^4^ cells/mL. As described in 2.5.3, the mouse peritoneal mesothelial cells and fibroblasts were stained with membrane fluorescent dyes Dil (red) and Dio (green) respectively. After the two kinds of cells were resuspended with DMEM complete culture medium, they were cocultured according to the proportion of 1:1 on the PRF film prepared earlier. According to the different culturing times, they were divided into four groups with three independent biological replicates in each group. After 0.5h, 1h, 2h, and 4h of culture, PBS was used to wash and remove the unattached cells respectively. The number and proportion of the two kinds of cells were observed with a laser-scanning confocal microscope.

### 2.7 The effect of PRF on the migration ability of mesothelial cells

The effect of PRF on the migration ability of mesothelial cells was detected by scratch assay and transwell migration assay.

#### 2.7.1 Scratch assay

A marker pen and ruler were used to draw straight lines at intervals of 0.5cm on the back of 6-well plates (Corning, USA) as marking points with 5 straight lines drawn for each hole. Mesothelial cells were digested and the cell concentration was adjusted to 5×10^5^ cells/mL so that cells were inoculated into the 6-well plates and cultured overnight in the cell incubator. After the cells grow and fuse to about 80%, use a 200μl pipette tip (Thermo Fisher Scientific, USA) to scratch along the central part of the well plate perpendicular to the marking line. Add mitomycin C (20μg/ml) to the serum-free medium to avoid the influence of cell proliferation on migration experiments. The appropriate concentration of PRF leachate was selected according to the CCK-8 assay. Cells were divided into a control group and a PRF group based on whether PRF was added to the well. Three independent biological replicates were assessed. The scratched areas were monitored under an inverted microscope (40×), and images were captured at 0h, 24h, and 48h to track cell migration. Use Image J software to analyze and calculate the scratch healing area of two groups of cells.

#### 2.7.2 Transwell migration assay

DMEM basic medium without FBS was selected to starve mesothelial cells and culture them in the cell incubator for 12-24h. Mesothelial cells were digested and the cell concentration was adjusted to 1×10^4^ cells/mL so that cells were inoculated into the 24-well plates (Corning, USA) and 200µL of cell suspension was added to the upper chamber of the Transwell plates (Corning, USA). The appropriate concentration of PRF leachate was selected according to the CCK-8 assay. 500µL of DMEM complete culture medium was added to the lower chamber of the control group and 500µL of PRF leachate was added to the lower chamber of the PRF group. Divide each group into two subgroups according to different cultivation times with three independent biological replicates in each subgroup. After 24h and 48h of cultivation, remove the upper chamber and fix the cells with 200uL of 4% paraformaldehyde (Solarbio, China), discard the fixative after 15 min. Incubate each chamber with 200uL of 0.1% crystal violet (Solarbio, China) at room temperature after washing with PBS. Discard the crystal violet after 10 min and wash with PBS until no crystal violet dye solution seeps out. Randomly record the number of migrating cells in 5 fields of view in each chamber using an inverted microscope (100×) and take the mean count.

### 2.8 Western Blot detects the effect of PRF on the MMT of mesothelial cells

According to whether PRF leachate was added or not, mesothelial cells were divided into the control group and the PRF group. After 48 hours, cells were collected and total protein was isolated from mesothelial cells using RIPA buffer containing protease and phosphatase inhibitors (Solarbio, China). The protein concentration was measured using the BCA protein assay kit (ThermoFisher Scientific, USA). Subsequently, equal amounts of protein samples were separated by 10% SDS-PAGE polyacrylamide gels and transblotted onto nitrocellulose membranes (BioRad, USA). After blocking with 5% bovine serum albumin at room temperature for 2 hours, the membrane was incubated overnight with the first antibody at 4 °C and then incubated with Alexa Fluor's second antibody at room temperature for 1 hour. Images were obtained using Odyssey CLx (LI-COR Biosciences, USA). Normalize the expression level of the protein to the expression level of GAPDH (Proteintech, 60004-1-lg, 1:200000 diluted, USA). Quantify protein bands using Image J software (NIH, Bethesda, MD, USA). The primary antibodies used include E-cadherin (Proteintech, 20874-1-AP, 1:1000 diluted, USA), vimentin (Bioss, bs-0756R, 1:1000 diluted, China), Snail2 (Proteintech, 12129-1-AP, 1:1000 diluted, USA), and the secondary antibodies used include Goal anti-rabbit antibody (680 anti-rabbit IgG, Yeasen, 33118ES60, 1:25000 diluted, China) and Goal anti-mouse antibody (790 anti-mouse IgG, Yeasen, 33219ES60, 1:25000 diluted, China).

### 2.9 The effect of PRF on the expression level of CR in mesothelial cells

Immunofluorescence, quantitative real-time polymerase chain reaction (qRT-PCR), and Western Blot were used to detect the effect of PRF on the expression level of CR in mesothelial cells.

#### 2.9.1 Immunofluorescence

Soak sterile cell slides in a 0.1mg/ml polylysine solution (Solarbio, China) for 5-10 min and place them in 12-well plates (Corning, USA) after air drying. Mesothelial cells were digested and the cell concentration was adjusted to 1×10^5^ cells/mL so that cells were inoculated into the 12-well plates. The appropriate concentration of PRF leachate was selected according to the CCK-8 assay. Divide cells into a control group and a PRF group based on whether PRF was added or not. Three independent biological replicates were assessed in each group. After culturing for 48h, the cells were fixed with 4% paraformaldehyde for 15 min and permeabilized with 0.3% TritonX-100 solution (Solarbio, China) for 30min as well as blocked by 5% donkey serum (Solarbio, China) at room temperature for 1 h. Add 1:100 diluted rabbit polyclonal anti-calretinin (CR) antibody (Bioss, bs-0062R, China) and incubate it overnight at 4 °C. On the second day, the CR antibody was retrieved and 1:500 diluted goat anti-rabbit IgG secondary antibody (Alexa Fluor 594, Abcam, ab150080, USA) was added to the cells. Incubate the antibody at room temperature for 1h (all subsequent operations were carried out in a dark environment). The nuclei were stained for 5 min with DAPI (Procell, China). The images of slides were then obtained using a fluorescence-inverted microscope (Shanghai, Cai Kang). Use Image J software to analyze images.

#### 2.9.2 Quantitative real-time polymerase chain reaction (qRT-PCR)

According to whether PRF leachate was added or not, mesothelial cells were divided into the control group and the PRF group. After 48 hours, cells were collected and total RNA was extracted from mesothelial cells using TRIzol reagent (Invitrogen, USA). According to the manufacturer's instructions, use PrimeScript ™ The RT kit (TaKaRa, Japan) transcribed the isolated RNA into cDNA. Perform qRT PCR on the BioRad real-time system using SYBR Green Master Mix (Thermo Fisher Scientific, USA). Normalize the expression level of genes to the expression level of GAPDH and calculate using the 2^-ΔΔCt^ method. All experiments were repeated. The primers used for qRT PCR are as follows:

CR, forward, 5'-AGTACACCCAGACCATACTACG-3', reverse, 5'-GAGGGCGTCCAGTTCATTCT-3'; GAPDH, forward, 5'-GGCACAGTCAAGGCTGAGAATG-3', reverse, 5'-ATGGTGGTGAAGACGCCAGTA-3'.

#### 2.9.3 Western blot

Refer to experiment 2.8 for details. The primary antibodies used include CR (Bioss, bs-0062R, 1:1000 diluted, China), and the secondary antibodies used include Goal anti-rabbit antibody (680 anti-rabbit IgG, Yeasen, 33118ES60, 1:25000 diluted, China).

### 2.10 Data analysis

Prism 9 (GraphPad Software, USA) and SPSS Statistics 26.0 (IBM software, USA) were used to perform the statistical analyses. The data were expressed as Mean ± Standard Deviation (M±SD). Two groups were compared using the student's t-test. Comparisons between three or more groups were performed using one-way Analysis of Variance (ANOVA) followed by the Bonferroni multiple comparison test. P < 0.05 were considered statistically significant in all results.

## 3. Results

### 3.1 Morphology and identification of primary mouse peritoneal mesothelial cells and fibroblasts

**Figure 2:**
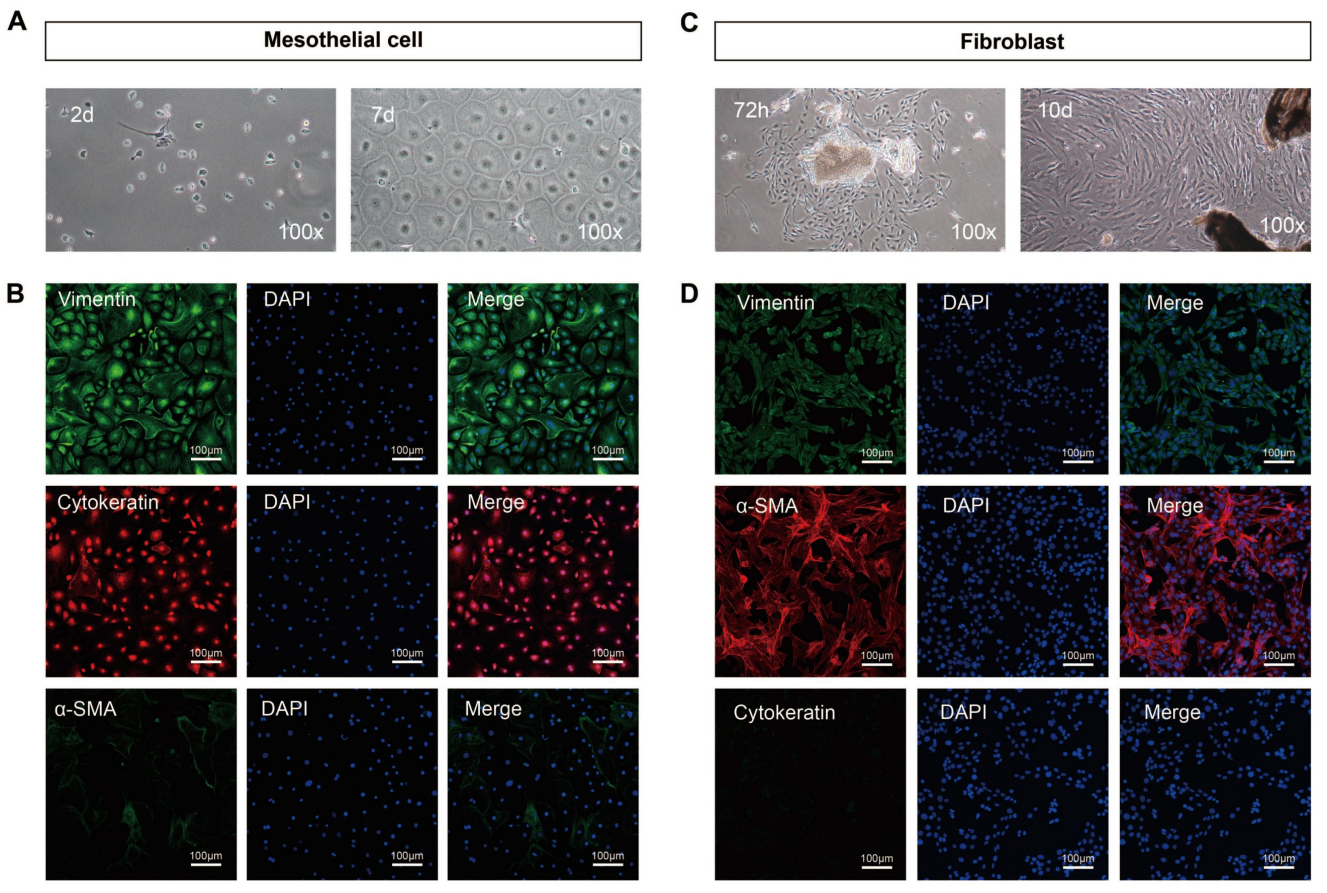
** Morphological characteristics and identification results of primary mouse peritoneal mesothelial cells and fibroblasts.** (A) The primary mesothelial cells became polygonal and gradually merged into the shape of pebbles or cobblestones after 24h and 7 days of cultivation (100×). (B) Immunofluorescence results showed that the isolated mesothelial cells positively expressed cytokeratin and vimentin but did not express α-SAM. (C) After 72h of cultivation, a small number of fibroblasts began to grow from the edge of the tissue block, presenting typical fibrous and elongated spindle shapes with overlapping growth. The fibroblasts gradually merged into the shape of a radial or fish cluster after growing for 10 days (100×). (D) Immunofluorescence results showed that the isolated fibroblasts expressed α-SAM and vimentin but did not express cytokeratin. Scale bars = 100 μm.

Under the optical microscope, primary mesothelial cells isolated from mice peritoneum exhibited suspended spherical shapes. After 24h of incubation, the cells adhered to the wall and gradually fused into a pebble or cobblestone shape at 5-7 days (100×) (Figure 2A). Cytokeratin and vimentin, two well-established markers of mesothelial cells, are positively expressed in mouse peritoneal mesothelial cells while α-SAM is negatively expressed (Figure 2B). After 72h of the adherent culture of the inoculated peritoneal tissue block, a small number of fibroblasts began to grow from the edge of the tissue block, presenting typical fibrous and elongated spindle shapes with overlapping growth. The fibroblasts gradually merged into the shape of a radial or fish cluster after growing for 10 days (100×) (Figure 2C). Immunofluorescence results indicated that the isolated fibroblasts expressed high levels of vimentin and α-SAM and almost no expression of cytokeratin (Figure 2D). Collectively, peritoneal mesothelial cells and fibroblasts were successfully isolated and purified.

### 3.2 PRF tends to promote the proliferation of mesothelial cells rather than fibroblasts

**Figure 3:**
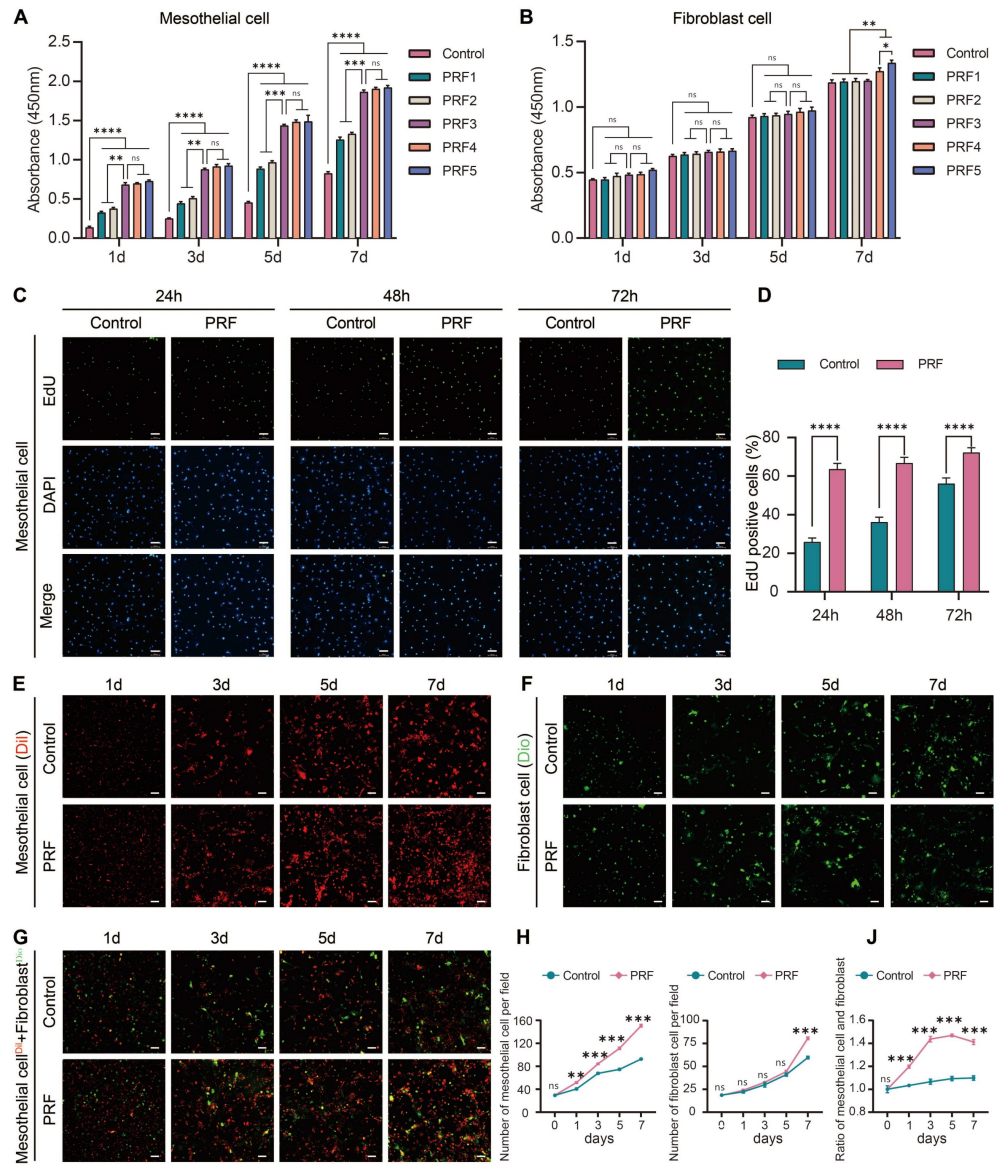
** PRF tends to promote the proliferation of mesothelial cells rather than fibroblasts.** (A) On the 1st, 3rd, 5th, and 7th days of cultivation, the OD values of mesothelial cells in the five groups of PRF were all higher than those in the control group, and the OD values of the PRF3, PRF4, and PRF5 groups were all higher than those of the PRF1 and PRF2 groups while there was no significant difference among the PRF three groups. (B) On the 1st, 3rd, and 5th day of fibroblast culture, there was no significant difference in OD value between the control group and the experimental group (PRF1, PRF2, PRF3, PRF4, and PRF5 groups) (*P*>0.05). On the 7th day of cultivation, only the OD values of PRF4 and PRF5 groups were higher than those of the control group, and the OD values of PRF5 were higher than PRF4. (C, D) The Edu assay showed that after treatment with 100% concentration of PRF leachate for 24, 48, and 72h, the percentage of Edu-positive cells (green) in the mesothelial cells of the PRF group at each time point was higher than that of the control group. (E, H) The results of the laser scanning confocal microscope showed that the number of mesothelial cells (Dil, red) in the PRF group was significantly higher than that in the control group on the 1st, 3rd, 5th, and 7th days of culture. (F, I). The number of fibroblasts in the PRF group was significantly higher than that in the control group (Dio, green) on the 7th day of cultivation. (G, J) When mesothelial cells (Dil, red) and fibroblasts (Dio, green) were cocultured according to the proportion of 1:1 on the 1st, 3rd, 5th, and 7th days, the mesothelial cells/fibroblasts ratio in the PRF group was higher than that in the control group at all time points. *P < 0.05, **P < 0.01, ***P < 0.001, ****P < 0.0001. Scale bars = 100 μm.

As detected by CCK-8 assays, the proliferation capacity of mesothelial cells treated with the PRF1, PRF2, PRF3, PRF4, and PRF5 were higher than those in the control group (*P*<0.0001). Moreover, treatment of mesothelial cells with PRF3, PRF4, and PRF5 for indicated time points resulted in elevated proliferation ability compared to those treated with PRF1 and PRF2 (*P*<0.01). However, there was no significant difference between the PRF3, PRF4, and PRF5 groups (*P*>0.05) (Figure 3A), indicating that PRF promotes mesothelial cell proliferation from the 1st day and could continue until the 7th day. PRF leachate with a concentration of over 60% had a stronger ability to promote mesothelial cell proliferation than the PRF leachate with concentrations of 20% and 40%, but no statistical difference between PRF leachate with concentrations of 60%, 80%, and 100% was detected. Therefore, PRF leachate with a concentration of over 60% was the appropriate concentration to promote mesothelial cell proliferation. On the 1st, 3rd, and 5th day of fibroblast culture, there was no significant difference in OD value between the control group and the experimental group (PRF1, PRF2, PRF3, PRF4, and PRF5 groups) (*P*>0.05). On the 7th day of cultivation, only the OD values of PRF4 and PRF5 groups were higher than those of the control group (*P*<0.01), and the OD values of PRF5 were higher than PRF4 (*P*<0.05) (Figure 3B), indicating that PRF only significantly promotes fibroblast proliferation on the 7th day. PRF leachate with concentrations of 80% and 100% could promote fibroblast proliferation and the ability of PRF leachate with concentrations of 100% to promote fibroblast proliferation was significantly greater than 80%. Therefore, PRF leachate with concentrations of 100% was the optimal concentration for promoting fibroblast proliferation. To avoid the influence of different concentrations of PRF leachate on cell proliferation, two types of cells were cultured with PRF leachate with concentrations of 100%. Edu assay was conducted on mesothelial cells after 24h, 48h, and 72h of cultivation. The results showed that the percentage of Edu-positive cells (green) in the PRF group at each time point was higher than that in the control group (*P*<0.0001) (Figure 3C, D), which further indicates that PRF can promote mesothelial cell proliferation. Meanwhile, the results of laser scanning confocal microscopy showed that the number of mesothelial cells (Dil, red) in the PRF group was significantly higher than that in the control group on the 1st, 3rd, 5th, and 7th days of cultivation (*P*<0.01) (Figure 3E, H). The number of fibroblasts (Dio, green) in the PRF group was significantly higher than that in the control group on the 7th day of cultivation (*P*<0.001) (Figure 3F, I). When mesothelial cells (Dil, red) and fibroblasts (Dio, green) were cocultured according to the proportion of 1:1 on the 1st, 3rd, 5th, and 7th days, the mesothelial cells/fibroblasts ratio in the PRF group was higher than that in the control group at all time points (*P*<0.001) (Figure 3G, J). Taken together, the effect of PRF on the proliferation of mesothelial cells starts earlier and lasts longer while the effect on the proliferation of fibroblasts starts later. Moreover, PRF tends to promote the proliferation of mesothelial cells rather than fibroblasts within 1-7 days.

### 3.3 PRF tends to promote the attachment of mesothelial cells rather than fibroblasts

**Figure 4:**
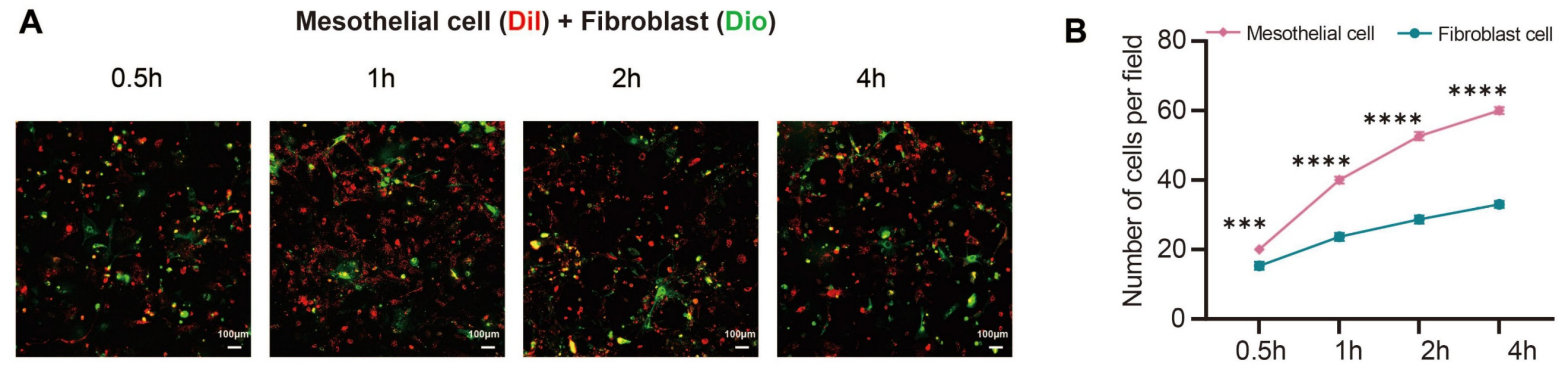
**PRF tends to promote the attachment of mesothelial cells rather than fibroblasts.** (A, B) The results of confocal microscopy showed that the number and proportion of mesothelial cells (Dil, red) were higher than those of fibroblasts (Dio, green) when two types of cells were seeded in equal proportions on the PRF film. ***P < 0.001, ****P < 0.0001. Scale bars = 100 μm.

The laser scanning confocal microscopy results showed when mesothelial cells (Dil, red) and fibroblasts (Dio, green) were seeded on the PRF membrane for 0.5h, 1h, 2h, and 4h, the number and proportion of mesothelial cells attached to the PRF membrane were higher than those of fibroblasts (*P*<0.001) (Figure 4A, B).

### 3.4 PRF promotes mesothelial cell migration

**Figure 5:**
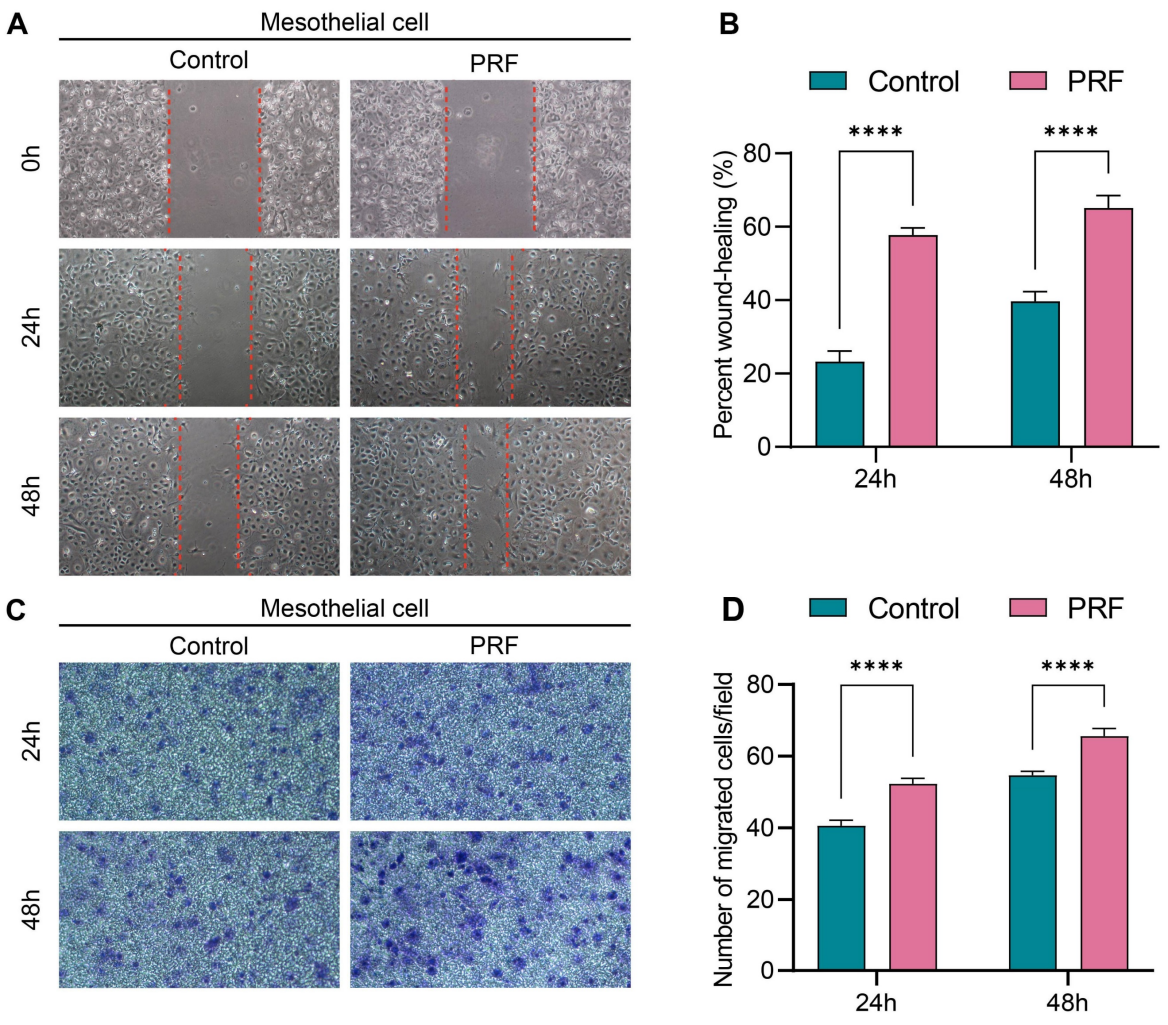
**PRF promotes mesothelial cell migration.** (A, B) The scratch assay results showed that the wound closure rates of the PRF group were higher than that of the control group at different time points. (C, D) The transwell migration assay results showed that the number of mesothelial cells migrating in the transwell chamber of the PRF group was higher than that of the control group at different time points. (100×), ****P < 0.0001.

The 100% concentration of PRF leachate was selected for cell migration assay according to the CCK-8 assay. Scratch assay results showed that the wound closure rates of mesothelial cells treated with PRF for 24 or 48h were significantly higher than that in control (*P*<0.0001) (Figure 5A, B). Similarly, the Transwell migration assay demonstrated that mesothelial cells treated with PRF for 24h or 48h exhibited significantly increased migration ability compared to the control group (*P*<0.0001) (Figure 5C, D). These results indicate that PRF promotes the migration ability of mesothelial cells.

### 3.5 PRF had a limited regulatory effect on the MMT of mesothelial cells

**Figure 6:**
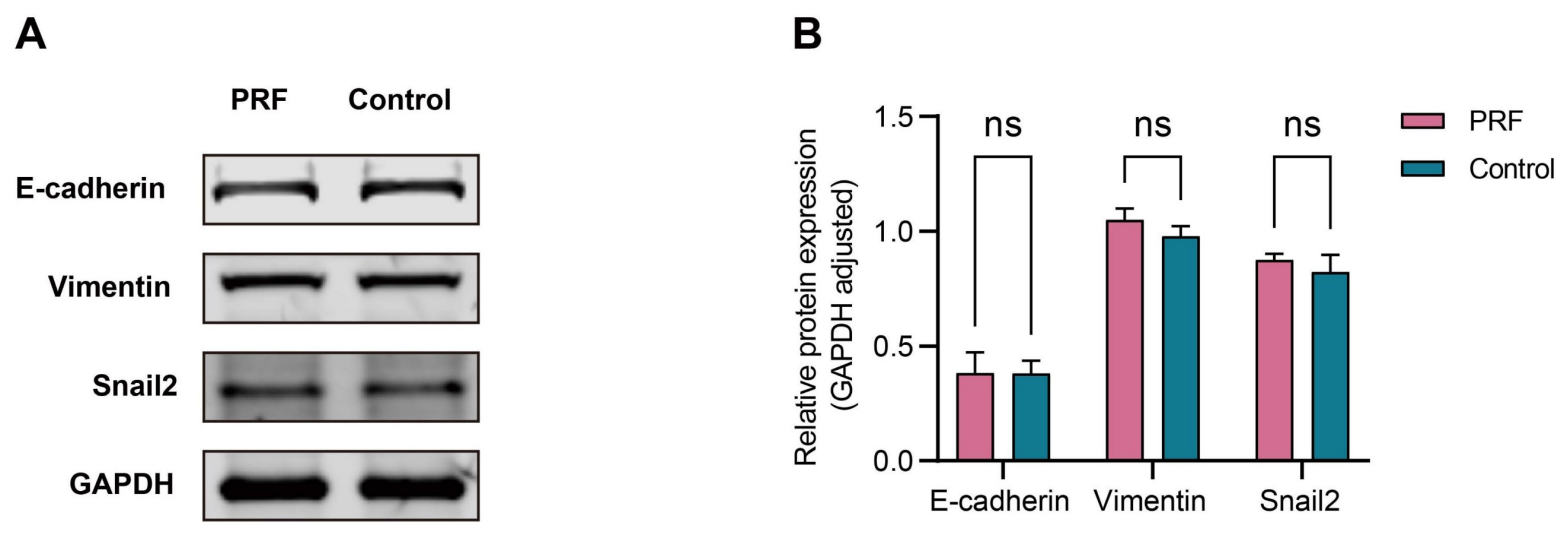
** PRF had a limited regulatory effect on the MMT of mesothelial cells.** (A) The expression levels of E-cadherin, vimentin, and Snail2 in mesothelial cells treated with PRF or not. (B) Quantitative analysis results showed no significant difference in the protein levels of E-cadherin, vimentin, and Snail2 between the PRF group and control group (*P* > 0.05).

The 100% concentration of PRF leachate was selected to detect the effect of PRF on the MMT of mesothelial cells according to the CCK-8 assay. The results of Western Blot showed that there was no significant difference in the expression levels of E-cadherin, vimentin, and Snail2 proteins in mesothelial cells between the PRF group and the control group after 48 hours (*P*>0.05) (Figure 6A, B).

### 3.6 PRF upregulates the expression level of CR in mesothelial cells

**Figure 7:**
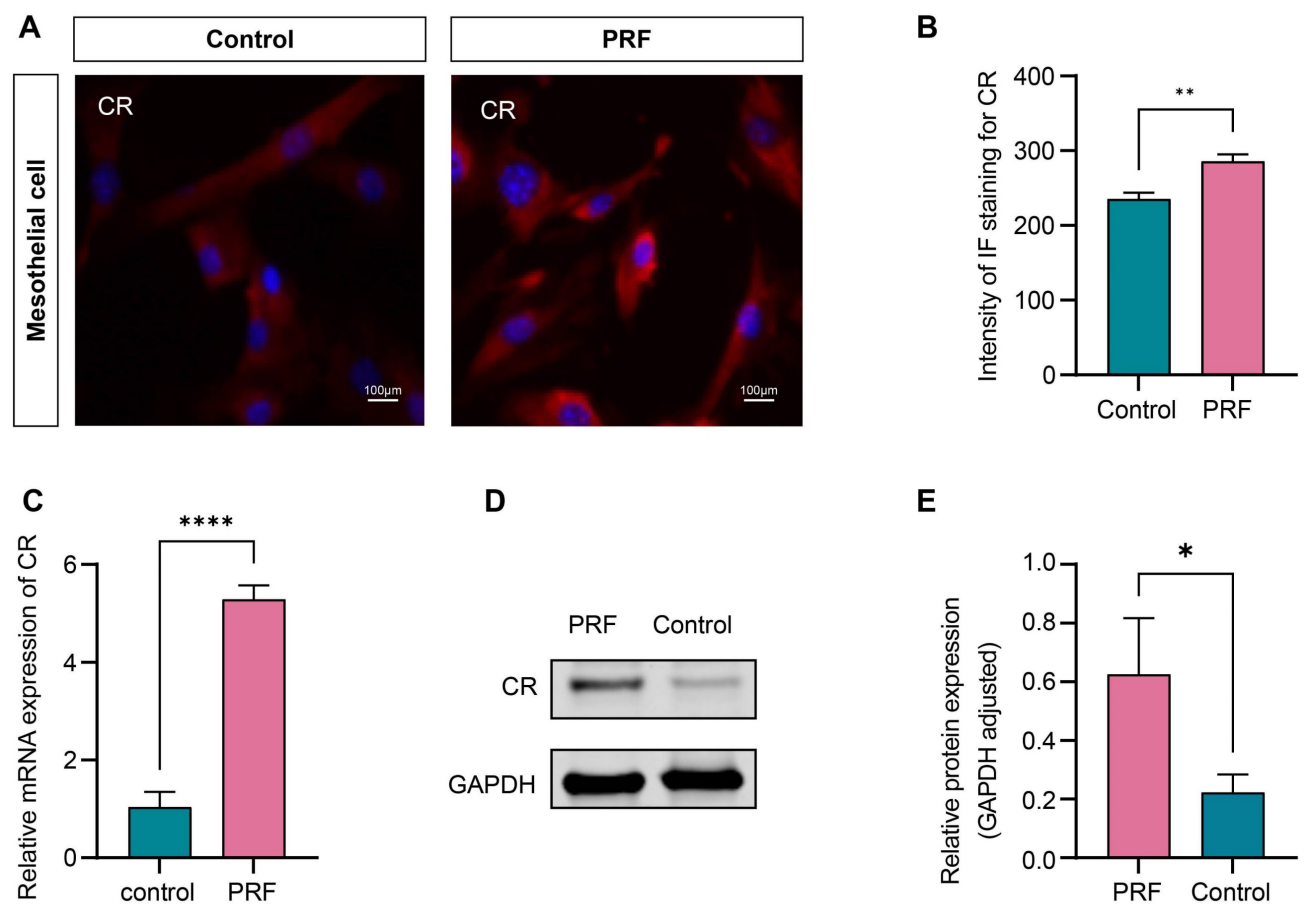
**PRF upregulates the expression level of CR in mesothelial cells.** (A) Representative immunofluorescence images of CR in mesothelial treated with PRF or not. (B) Quantification of the fluorescence intensity of CR in the PRF and control groups. (C) The qRT-PCR showed that the mRNA expression level of CR in the PRF group was significantly higher compared with the control group. (D, E) Western Blot's results showed that the CR protein expression level in the PRF group was significantly higher compared with the control group. *P < 0.05, **P < 0.01. ****P < 0.0001. Scale bars = 100 µm.

The 100% concentration of PRF leachate was selected to detect the effect of PRF on the expression level of CR in mesothelial cells according to the CCK-8 assay. The results of immunofluorescence showed that the fluorescence intensity of CR in the PRF group significantly increased compared to the control group after 48 hours (*P*<0.01) (Figure 7A, B). qRT-PCR and Western Blot were performed to further validate the conclusion. The results of qRT-PCR showed that compared with the control group, the gene expression level of CR mRNA in the PRF group was significantly higher (*P*<0.0001) (Figure 7C). The results of Western Blot showed that the expression level of CR protein in the PRF group was significantly higher compared with the control group(*P*<0.05) (Figure 7D, E).

## 4. Discussion

Abdominal adhesions refer to abnormal fibrous connections formed between abdominal organs, omentum, and abdominal wall due to injury and inflammation 33. Abdominal surgery is the main cause of postoperative intestinal adhesions, with about 90% of postoperative intestinal obstruction caused by abdominal adhesions.

Sometimes the abdominal adhesions require surgical treatment but reoperation can also cause new adhesions, which form a vicious cycle 1,5,34. To prevent postoperative abdominal adhesions, scholars have conducted extensive research. At present, the research on new anti-adhesion materials is a hot topic in preventing abdominal adhesions. In recent years, commonly used materials such as sodium hyaluronate and hydrogel isolate the damaged serosa surface through the physical barrier function to make the wound heal itself, inhibit the proliferation of fibroblasts, reduce the formation of fibrin, and promote the dissolution of fibrin, to achieve a certain effect of preventing adhesion 35,36. However, the existing materials for preventing abdominal adhesions still fall short in terms of safety and/or effectiveness 12,37. The PRF used in this study was prepared from the homologous animal blood. In humans, if PRF is prepared from autologous blood, the raw materials are simple and readily available and theoretically without immune reactions, making it more suitable for patients and helping to reduce medical costs and alleviate patient burden. In addition, the three-dimensional network structure of PRF has large pores and good elasticity, which is conducive to the retention of cytokines and cell growth, thereby facilitating the transport of nutrients and oxygen. This is the structural basis for PRF to uniformly release growth factors for a long time and prolong the effective action time 20. Our previous research found that PRF can prevent postoperative intestinal adhesions 26. The results of this study also confirm that PRF can promote rapid proliferation of mesothelial cells from 1-7 days. These studies suggest that the proliferation of mesothelial cells promoted by PRF plays a key role in preventing intestinal adhesions. When PRF is applied to the site of peritoneal injury, it promotes rapid proliferation of mesothelial cells covering the top layer of the peritoneum firstly, forming a complete and smooth layer of mesothelial cells that covers the surface of the peritoneal injury.

At present, the materials used in clinical practice for preventing adhesions mainly focus on physical barriers and reducing inflammatory reactions, neglecting the importance of rapid and effective promotion of peritoneal repair to prevent intestinal adhesions. The peritoneum is covered by a thin layer of mesothelial cells. Below the mesothelial cells, the basement membrane and stromal subcutaneous tissue can be seen, including collagen fibers, blood vessels, and fibroblasts 11. The integrity and normal function of mesothelial cells are crucial for preventing peritoneal adhesion. When the peritoneum is damaged, mesothelial cells in the peritoneum may undergo shedding, necrosis, or apoptosis. The reactive proliferation of mesothelial cells is a characteristic of peritoneal healing. On the first to third day of peritoneal injury repair, neutrophils, monocytes, macrophages, and mesothelial cells can be found on the wound surface, and then mesothelial cells gradually become dominant on the surface of the wound over the next 4-7 days. During peritoneal repair, extensive mesothelial cell islands are formed on the surface of the damaged area, which continuously fuse with each other to promote wound healing. Both large and small wounds can heal within 5-7 days postoperatively 13. As an important component of body injury repair, the fibrinolytic system can lead to abdominal adhesion by inducing excessive deposition of fibrin 38. Mesothelial cells secrete tissue-type plasminogen activator (t-PA) and plasminogen activator inhibitor (PAI), and the balance between t-PA and PAI determines the activity of plasminogen activator 39,40. During the repair process of peritoneal injury, the decrease of t-PA and the increase of PAI can disrupt the balance between t-PA and PAI, leading to a decrease in fibrinolytic enzyme synthesis and fibrin cannot be dissolved early, which promotes adhesion formation. If the function and quantity of mesothelial cells decrease, fibroblasts gradually proliferate and attach to fibrin that has not been dissolved in time, producing collagen and forming adhesive fibrous tissue 13,41. On the contrary, adhesion can be reduced if mesothelial cells proliferate rapidly, accelerating the coverage of peritoneal mesothelial cells at the site of peritoneal injury and relatively inhibiting fibroblast proliferation, indicating that promoting mesothelial cell rapid proliferation of damaged peritoneum becomes the key to preventing intestinal adhesion. The roles of mesothelial cells and fibroblasts in the healing process of peritoneal injury are different. The proliferation of mesothelial cells may alleviate adhesions while the proliferation of fibroblasts may aggravate adhesions. This is also why these two types of cells were selected as the research subjects for this study. The results showed that PRF tends to promote the proliferation of mesothelial cells rather than fibroblasts. We speculate that during the formation of abdominal adhesions, these two types of cells compete for proliferation, and mesothelial cells have stronger proliferation ability than fibroblasts. Mesothelial cell islands can cover the surface of the wound in the early stage of peritoneal injury, thereby reducing adhesions. Our previous research on mice with intestinal adhesions demonstrates that PRF can prevent and alleviate intestinal adhesions by promoting the rapid proliferation of mesothelial cells, relatively inhibiting fibroblast proliferation and infiltration of inflammatory cells 26.

Research has shown that PRF can promote the attachment of various cells such as osteoblasts 42, as well as the migration and proliferation ability of fibroblasts 43. However, there is currently no relevant study on the effect of PRF on the proliferation and migration ability of mesothelial cells. Based on our previous research, this study focuses on comparing the effects of PRF on the proliferation and adhesion abilities between mesothelial cells and fibroblasts, and further exploring the mechanism of PRF in preventing intestinal adhesions. The results showed that PRF leachate with concentrations above 60% had a promoting effect on the proliferation of mesothelial cells, and there was no statistical difference in the ability of PRF leachate with concentrations of 60%, 80%, and 100% to promote the proliferation of mesothelial cells, indicating that low concentrations of PRF are not sufficient to promote the maximum proliferation potential of mesothelial cells. When the required concentration of PRF can already promote the maximum proliferation efficiency of mesothelial cells, higher concentrations of PRF no longer increase their proliferation effect. PRF promotes the proliferation of mesothelial cells from day 1 and can last until day 7, while PRF only significantly promotes the proliferation of fibroblasts on day 7, indicating that the effect of PRF on mesothelial cell proliferation starts earlier and lasts longer and the effect on fibroblast proliferation starts later. Moreover, PRF tends to promote the proliferation of mesothelial cells rather than fibroblasts within 1-7 days. In addition, we also found that PRF has a stronger ability to promote the attachment of mesothelial cells rather than fibroblasts. Moreover, PRF can also promote the migration of mesothelial cells. Research has found that in the early stage of abdominal adhesions, the enhanced proliferation and migration ability of mesothelial cells covering the top layer of the peritoneum can regenerate and rebuild the damaged mesothelial cell layer, effectively inhibiting the formation of mature adhesions 11. A recent study showed that mesenchymal stem cells promote the healing of peritoneal wound and prevent peritoneal adhesion, which is also related to the proliferation and migration of mesothelial cells 44. Usually, the healing of peritoneal wounds takes about a week after injury 13, while PRF promotes rapid proliferation of mesothelial cells from 1-7 days in the early stages of peritoneal injury, forming a complete and smooth layer of mesothelial cells. At the same time, by promoting rapid migration and attachment of mesothelial cells, PRF with a large number of mesothelial cells "preemptively occupies" the site of peritoneal injury and relatively inhibits fibroblast proliferation until the serosal injury surface fully heals, thereby preventing and reducing intestinal adhesions. Its mechanism is completely different from the traditional mechanism of other materials for preventing intestinal adhesions.

The mechanism of abdominal adhesions is complex and inconclusive. Coagulation, reduced fibrinolysis, MMT, macrophage superaggregates, NETs, fibroblasts, mesothelial cells, and ECM generation may all be involved in it 45. The balance between t-PA and PAI is critical for maintaining the stability of the fibrinolytic system. During the peritoneal injury repair process, a decrease in t-PA and an increase in PAI can lead to reduced synthesis of fibrinolytic enzymes. Consequently, the untimely dissolution of fibrin will lead to excessive deposition and subsequent adhesion formation. Mesothelial cells can promote fibrinolysis by secreting increased levels of t-PA 46. Our studies have demonstrated that PRF can enhance the proliferation of mesothelial cells. It is currently unclear whether PRF can alleviate intestinal adhesions by regulating the fibrinolytic system. NETs are structures composed of DNA, histones, and granule proteins, released by neutrophils 47. NETs contribute to adhesion formation by promoting fibroblast activation, ECM remodeling, and angiogenesis 9. Our preliminary animal experiments indicate that PRF may inhibit the infiltration of inflammatory cells 26. Further research is needed to to determine whether PRF has an impact on macrophage superaggregates and NETs. Sandoval *et al.* were the first to establish that the MMT of peritoneal mesothelial cells serves as a major source of myofibroblasts in abdominal adhesions, emphasizing that MMT significantly contributes to the development of pathological adhesions 48. In this study, we observed that PRF has a limited effect on regulating MMT. The proliferation and migration of mesothelial cells were enhanced as early as day 1 following PRF treatment. We hypothesize that the increased migration and proliferation of mesothelial cells induced by PRF facilitates their recruitment to the injury site, thereby accelerating the mesothelial repair process and allowing the peritoneal injury surface to heal prior to the onset of MMT. Its mechanism is completely different from the traditional mechanism of other materials for preventing intestinal adhesions.

But how does PRF promote mesothelial cell proliferation? Research has shown that growth factors, Wnt proteins, and β-catenin are all associated with cell proliferation 49. Growth factors belong to the receptor tyrosine kinase family and can promote cell proliferation through the Wnt signaling pathway 50. The classic Wnt pathway, also known as the Wnt/β-catenin pathway, is widely involved in physiological processes such as cell proliferation, activation, and migration, and plays a key role in maintaining homeostasis in the human body 51. In addition, the Wnt/β-catenin pathway also plays a role in regulating the physiological functions of peritoneal mesothelial cells 52. Because of abundant and multiple growth factors, PRF also plays a role in regulating the proliferation and differentiation of bone marrow mesenchymal stem cells through the Wnt pathway 53. The coding gene Calb2 of CR, as a direct target of β-catenin, may play an important role in the Wnt/β-catenin pathway affecting the activity of the thalamocortical circuit 54. Research showed that CR is positively correlated with mesothelial cell proliferation 27. More importantly, our results showed that PRF could upregulate the expression level of CR in mesothelial cells. Therefore, we speculate that PRF may upregulate CR through the Wnt/β-catenin pathway, promote mesothelial cell proliferation, and accelerate peritoneal healing, thereby preventing and reducing intestinal adhesions. As is well known, a complete and smooth mesothelial cell layer is crucial for preventing intestinal adhesions, but the mechanism by which PRF prevents intestinal adhesions is still unclear and requires further research. In the future, we will focus on the Wnt/β-catenin signaling pathway to further investigate the role and mechanism of PRF in promoting mesothelial cell proliferation and preventing postoperative abdominal adhesions. It is expected to provide new strategies for the clinical prevention of postoperative intestinal adhesions and lay a theoretical foundation for the development of new materials or drugs for preventing intestinal adhesions.

## 5. Conclusions

We first applied PRF for the prevention of intestinal adhesions and studied its mechanism. The ability of PRF to promote the proliferation and attachment of mesothelial cells is more significant than that of fibroblasts. In the early stage of intestinal adhesion formation, PRF may promote rapid proliferation of mesothelial cells by upregulating CR expression. Meanwhile, a complete and smooth layer of mesothelial cells is formed by promoting the rapid migration and attachment of mesothelial cells. PRF with a large number of mesothelial cells "preemptively occupies" the site of peritoneal injury, while relatively inhibits fibroblast proliferation. PRF plays an important role in preventing intestinal adhesion, and its mechanism is obviously different from that of traditional anti-adhesion materials.

## Abbreviations

PRF: platelet-rich fibrin
CR: calretinin
MMT: mesothelial-mesenchymal transition
ECM: extracellular matrix
NETs: Neutrophil extracellular traps
TGF-β: transforming growth factor-β
VEGF: vascular endothelial growth factor
PDGF: platelet-derived growth factor
EGF: epidermal growth factor
α-SAM: Alpha smooth muscle actin
t-PA: tissue-type plasminogen activator
PAI: plasminogen activator inhibitor
